# Efficacy and Safety of Deracoxib for the Control of Postoperative Pain and Inflammation Associated with Dental Surgery in Dogs

**DOI:** 10.5402/2011/593015

**Published:** 2012-01-23

**Authors:** Stephen E. Bienhoff, Eric S. Smith, Linda M. Roycroft, Elizabeth S. Roberts, Larry D. Baker

**Affiliations:** ^1^Department of New Product Development, Novartis Animal Health US, Inc., Greensboro, NC 27408, USA; ^2^Northgate Veterinary Dentistry, Decatur, IL 62526, USA

## Abstract

The efficacy and safety of deracoxib administered at 1-2 mg/kg/day for 3 days was assessed for the control of postoperative pain and inflammation associated with dental surgery in dogs. Client-owned dogs scheduled for dental extractions were premedicated with butorphanol and randomly assigned to receive either deracoxib (*n* = 31) or placebo (*n* = 31) preoperatively and again once daily for 2 additional days. Dogs were evaluated prior to and after surgery using a modified Glasgow Composite Pain Scale (mGCPS). Dogs could be rescued at any time if they scored ≥4 on the mGCPS or in cases of obvious discomfort. Rescued dogs were considered treatment failures for determining treatment response and were removed from the study. Of the 62 dogs enrolled, 57 were usable for the efficacy analyses and all were assessed for safety. Four of 27 deracoxib-treated dogs (14.8%) were rescued compared to 20 of 30 placebo dogs (66.7%) (*P* = 0.0006). Deracoxib-treated dogs also had numerically lower mGCPS scores. Eight of 31 deracoxib dogs (26%) had adverse events reported compared to 6 of 31 placebo dogs (19%). Results indicate perioperative administration of deracoxib to dogs at 1-2 mg/kg/day for 3 days significantly improves analgesia after dental surgery.

## 1. Introduction

Historically, postoperative pain management received little attention in veterinary practice. However, pain management is increasingly recognized by veterinarians as the standard of care for all types of surgeries [1–5]. In addition to promoting the well-being of the surgical patient, controlling postoperative pain and inflammation facilitates the healing process and helps avoid the development of chronic pain [6]. Dental extractions often involve the exposure and removal of bone in the mouth that requires pain management during and after surgery.

Measuring pain in animals is difficult and assessing oral pain can be especially challenging since pain may be masked by the animal and not readily evident to the observer. The Glasgow Composite Pain Scale (GCPS) is a composite scale generally accepted for assessing pain in dogs in a hospital setting based on observations of behavior [7, 8]. The GCPS is not specifically designed to assess oral pain associated with dental surgery. However, it does provide a basis for developing a pain scale that can be used to assess dental surgical pain in the dog when modified accordingly.

Because of their beneficial effects, nonsteroidal anti-inflammatory drugs (NSAIDs) administered alone or in combination with other analgesic drugs are being used more frequently by veterinarians for controlling postoperative pain and inflammation [9–11]. Depending on the patient's health, a multimodal approach to pain management has been recommended, including combinations of opioids, NSAIDs, local anesthetics, and dissociative drugs [12]. Indeed, when combined with opioids, NSAIDs have been shown to be an important pain management tool after invasive maxillectomy or mandibulectomy associated with removal of oral neoplasms in dogs [13]. Moreover, a better understanding of the phenomenon of hyperalgesia or “wind-up” pain has led to the practice of administering NSAIDs prior to surgery. Studies investigating the timing of NSAID administration indicate administration prior to induction of anesthesia ensures optimal postoperative pain control [11, 14–17].

Deracoxib (Deramaxx Chewable Tablets, Novartis Animal Health US, Inc., Greensboro, NC), an orally administered cyclooxygenase-2 (COX-2) selective NSAID [18–20], is widely used in dogs for the control of pain and inflammation associated with orthopedic surgery. In humans, studies suggest that selective COX-2 inhibitors may be more effective than opioid-containing analgesics for managing dental surgical pain [21]. As a COX-2 selective NSAID approved for use in dogs, deracoxib is a good candidate for investigating the efficacy of an NSAID for dental pain management.

In this study, we hypothesized that dogs receiving perioperative administration of deracoxib along with butorphanol as a preanesthetic would have superior analgesic and anti-inflammatory effects when compared to dogs receiving butorphanol and a placebo as measured by a “success/failure” outcome based on a modification of the GCPS scoring system.

## 2. Materials and Methods

### 2.1. Animals

Client-owned dogs of various breeds and gender that were at least 4 months of age, weighed at least 6.3 kg, and were scheduled for dental extractions were enrolled in the study. Dogs were excluded if they were dehydrated, on concomitant diuretic therapy, suffering from chronic painful conditions, had uncontrolled endocrine or systemic disorders, or had existing renal, cardiovascular, hepatic, or gastrointestinal tract dysfunction.

The study protocol was reviewed and approved by the Novartis Animal Health US, Inc. Institutional Animal Care and Use Committee. Each dog owner was fully informed of the details of the study, and a signed informed consent was received prior to their dog being enrolled in the study. The decision to conduct the study with a preanesthetic analgesic (butorphanol) administered once to all dogs in an “add-on” study design where only half the dogs received deracoxib treatment, unless rescued from the study, was carefully weighed between the scientific merit of the study and the potential pain experienced by the animals. Steps were taken to minimize, as much as possible, the discomfort associated with the lack of perioperative NSAID treatment for dogs receiving the placebo. Therefore, the “add-on” design, along with frequent observations and immediate rescue, was chosen as the most practical way to ascertain the effectiveness of the drug while minimizing the discomfort experienced by the patient. Dogs were evaluated at specific and frequent time points during the study and pain assessors were advised to rescue a dog at any time, either during specified evaluation time points or any time in between, if a dog appeared to experience discomfort, thus minimizing the amount of pain experienced by the patient.

### 2.2. Study Design

The study was a prospective, randomized, blinded, placebo-controlled, multicenter field study. Practitioners at four veterinary clinics located in the US enrolled dogs presented for dental procedures that included one or more surgical extractions of the canine tooth, maxillary 4th premolar, maxillary 1st molar, and mandibular 1st molar where the procedure involved creation of a gingival flap, sectioning as necessary, removal of bone, and closure of the surgical site. The goal was to enroll 60 usable cases for efficacy and safety evaluations. Eligible dogs were assigned unique case identification codes and were assigned to either the deracoxib or placebo control groups using a randomization list generated by a statistician. Blinding was maintained by separation of function. Individuals responsible for dosing were not allowed to participate in clinical assessments or recording of response data. All other individuals were blinded to treatment assignments and were not allowed to participate in dosing of animals. Dogs were hospitalized at the clinic throughout the study and housed individually in a quiet location so that accurate behavioral assessments could be made.

### 2.3. Modified Glasgow Composite Pain Scale (mGCPS)

The GCPS is a composite scale for assessing pain in dogs in a hospital setting based on observations of behavior where a categorical score is assigned within each behavior category based on the severity of the behavior or response exhibited by the dog [7, 8]. The GCPS behavior categories include vocalization, attention to wound area, mobility, response to touch, demeanor, and posture/activity. For this study, the GCPS was modified (mGCPS) to reflect and measure behaviors observed when assessing dogs experiencing pain associated with dental surgery. Eating was also assessed, but was not part of the mGCPS system nor was it included when summing mGCPS scores for determining pain intervention. Modifications to the GCPS scoring system (removal of “attention to wound area” and “mobility”) and the exclusion of “eating” as a pain measurement category were based on consultations with board-certified veterinary dentists. The mGCPS behavior categories that were used in this study are summarized in Table 1.

### 2.4. Experimental Procedures

A preenrollment visit was conducted to establish patient eligibility. During the initial visit and after receiving the owner's informed consent, the practitioner performed a preenrollment physical examination and collected blood and urine samples for hematology, serum chemistry, and urinalysis. A dog was considered eligible for enrollment in the study if the physical examination and clinical pathology results were considered acceptable by the practitioner. Dogs were brought to the clinic the morning of the dental surgery and a baseline pain evaluation using the mGCPS was done after the patient had acclimated to the clinic for a minimum of 2 h and prior to administration of any preanesthetics. Deracoxib or placebo (Pet-Tabs, Virbac Animal Health, Inc., Fort Worth, TX) was administered orally at least 1 h prior to surgery and before preanesthetics were administered, and again daily for two additional days if the dog was not removed from the study. The deracoxib dose of 1-2 mg/kg administered once daily was based on the approved osteoarthritis dose. The plasma terminal elimination half-life of deracoxib is approximately 3 h; however, clinical effectiveness is observed for a longer duration thus allowing for once daily dosing [22].

The investigator was instructed to premedicate the dog prior to surgery with butorphanol once at a dose of approximately 0.2–0.5 mg/kg administered IV or IM. Any combination of available products, with the exception of another NSAID, corticosteroids, opioids, ketamine, xylazine, medetomidine, dexmedetomidine, tiletamine/zolazepam, and local anesthetics, was used to facilitate a smooth induction, maintenance, and recovery from anesthesia. Dental extractions of the canine tooth, maxillary 4th premolar, maxillary 1st molar, and mandibular 1st molar were done according to acceptable veterinary dental procedures that included the creation of a flap, sectioning as necessary, removal of bone, and closure of the surgical site. Investigators ensured that all study participants were adequately hydrated prior to and during surgery, including the administration of fluid therapy during dental procedures. Except for cases where intervention therapy was needed, only routine preventative care was permitted while the dog was on study. All other treatments that might affect pain assessments were prohibited.

To ensure interpretative consistency, a single person experienced in evaluating patients for pain in a clinical setting was designated at each clinic to evaluate behavior in response to pain using the mGCPS. This person was blinded to treatment and conducted all mGCPS assessments at that clinic. Prior to study initiation, the pain assessor at each site was thoroughly trained in the use of the mGCPS scoring system through descriptive examples and discussions on how the scoring system assigns scores to the observed behaviors. The assessor was instructed to first observe the dog's behavior from a distance so as not to disturb the dog. Then the assessor was instructed to gradually increase their interaction with the dog, including removing the dog from the kennel to allow the dog to move around and manipulation of the surgical site. Based on the response of the dog to the interactions with the pain assessor, each of the four behavior categories was then scored.

Dogs were removed from the study if they had an mGCPS score ≥4, if the assessor determined at any time that pain intervention therapy was needed due to discomfort of the dog, or if the dog had a serious adverse event. Pain assessments were conducted prior to administration of preanesthetics and postsurgically at 90 min, 3 h, 5 h, and 8 h (±15 min) after extubation. Additional assessments occurred at 2 h and 8 h (±15 min) after drug administration the first day after surgery and again at 2 h (±15 min) after drug administration the second day after surgery. Subsequent drug administrations were targeted for 24 and 48 h after the first administration the day of surgery. If intervention therapy was necessary, blood and urine samples were collected for clinical pathology, an exit physical examination was performed, body weight was determined, and the dog was removed from the study. Dogs removed from the study were considered treatment failures and received alternative pain intervention at the discretion of the veterinary practice according to their pain management protocols. Dogs that were removed from the study were subsequently monitored an additional 24 h for adverse clinical signs and to determine if additional pain medication was needed. The owner of each dog enrolled in the study was called 3 to 10 days after discharge from the study to inquire about the overall condition of their dog and any abnormal clinical signs reported by the owner were recorded as adverse events.

Any observation that was unfavorable and unintended and occurred after drug administration to the dog was considered an adverse event, whether or not the observation was considered to be product related. Investigators made a determination of whether the adverse event was medically “serious” or “not serious.” A “serious” adverse event required active medical intervention and was considered by the investigator to be clinically significant whereas “not serious” adverse events resolved without additional treatment.

### 2.5. Statistical Analyses

Treatment response using this study design was unknown for power calculations prior to the start of the study; however, a target enrollment of 60 dogs (30 per group) with a standard deviation range between 0.34 and 0.48 would yield over 80% power to detect a difference of 40% or greater was considered sufficient to adequately assess efficacy and safety. All analyses were performed using statistical software (SAS/STAT software Version 9.1.3, SAS Institute Inc., Cary, NC). The deracoxib-treated group was compared to the placebo control group on a success/failure basis using the individual dog as the experimental unit. Treatment failure was defined as the need to remove a dog from the study due to pain intervention therapy or because of a serious adverse event. Superiority was established by a reduction in the proportion of rescues in the deracoxib-treated group compared to the placebo control group using GLIMMIX. Survival analyses (LIFETEST and TPHREG) were also performed. The mGCPS categorical variables Demeanor, Posture/Activity, Response to Touch, Vocalization and the sum of the mGCPS variables measured the day of surgery (90 min to 8 h after extubation) were analyzed using GLIMMIX. Eating was also analyzed the day of surgery, but was not included in the sum of the mGCPS variables. Last Observation Carried Forward (LOCF) was used for missing values of any dog rescued from the study prior to completing all observations the day of surgery. Categorical variables were not analyzed any day after surgery as the LOCF assumption, that the animal's response remains unchanged, becomes weaker the longer the assessment times are from the time of surgery. Hematology, serum chemistry, and urinalysis determined prior to treatment and at study exit were compared using analysis of covariance (ANCOVA) with the pretreatment value used as a covariate. Individual clinical pathology parameters were classified as “low,” “normal,” or “high” when compared to a laboratory-provided normal range for that parameter and shift tables were created for comparisons. Body weight changes from measurements taken prior to treatment and at study exit were analyzed using analysis of variance (ANOVA). All hypotheses were tested at a two-sided 0.05 level of significance.

## 3. Results

Four study sites enrolled 62 dogs of which 57 were usable for the efficacy analyses, 27 in the deracoxib-treated group and 30 in the placebo control group. Five cases (three in the deracoxib group and two in the placebo control group) were excluded due to data recording errors, protocol deviations, or cases enrolled that were later determined not to need extractions. All 62 dogs enrolled in the study were included in the demographic summaries and safety analyses. Breeds enrolled included various mixed and pure-breed dogs. The mean (±SD) age was 8.4 ± 3.4 years (range, 1.5 to 16.4 years), and mean body weight was 20.7 ± 11.0 kg (range, 6.3 to 49.4 kg). There were four (6%) intact females, 31 (50%) spayed females, one (2%) intact male, and 26 (42%) neutered males enrolled in the study. No significant differences were found when age, breed, sex, pretreatment body weight, concomitant medications administered, or physical examination findings were compared between treatment groups. In addition to butorphanol administered as a preanesthetic, protocols included the use of diazepam or atropine sulfate as a preanesthetic followed by propofol for induction and isoflurane administered in oxygen for anesthetic maintenance. Pain intervention medication, used individually or in combination, included the use of buprenorphine (two practices), hydromorphone (two practices), butorphanol (one practice), and tramadol (two practices) as rescue treatments.

To limit enrollment bias, dogs were enrolled as they were presented to the practitioner, regardless of the tooth to be extracted or the number of teeth to be removed, and assigned to treatment group using a computer-generated randomization list. This prevented the “picking” of extraction cases for a treatment group and minimized bias as much as possible. Extractions were randomized relatively well between the treatment groups, although there were more multiple extractions randomized to the deracoxib group (eight dogs) compared to the placebo group (four dogs). However, the difference was not statistically significant. There also were more molar extractions randomized to the deracoxib group (18 dogs) compared to the placebo group (10 dogs), but the difference was again not statistically significant. Canine and fourth premolar extractions made up the majority of cases and were more evenly randomized between the two treatment groups.

**Figure 1:**
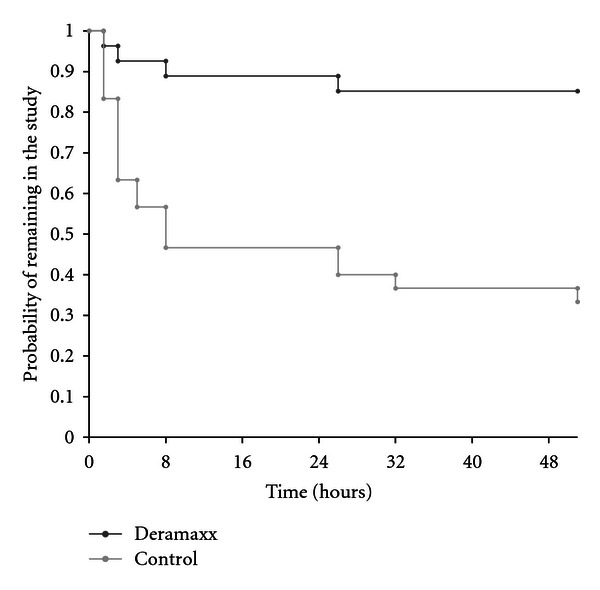
Kaplan-Meier study exit curves from pre-surgery to day 2 (50 h) after which time all remaining dogs were removed from the study. There were significant differences in both the Cox-Tarone (*P* = 0.0001) and Gehan-Breslow (*P* = 0.0009) tests between the deracoxib treatment group and the placebo control group, in favor of the deracoxib group.

Perioperative administration of deracoxib for 3 days in addition to a one-time preanesthetic administration of butorphanol significantly (*P* = 0.0006) reduced the need for rescue therapy and removal of the dog from the study when compared to a one-time administration of butorphanol along with a placebo. Only four of 27 dogs (14.8%) in the deracoxib-treated group required pain intervention compared to 20 of 30 dogs (66.7%) in the placebo control group (Table 2). Three of the four deracoxib treatment failures were rescued the day of surgery whereas the remaining dog was rescued the day after surgery. Of the 20 negative control dogs that were rescued, 16 were rescued the day of surgery, three were rescued the day after surgery, and the remaining dog was rescued two days after surgery. The specific outcome encountered in this study (*n* = 27 and 30, mean difference of 52%, SDs of 0.36 and 0.48) yielded over 90% power for detecting a difference. When survival analyses were used to assess treatment response, both the Cox-Tarone and Gehan-Breslow tests were significant (*P* = 0.0001 and 0.0009, resp.; Figure 1).

Overall baseline mGCPS scores were numerically similar between treatment groups prior to dental surgery. However, mean mGCPS scores were numerically lower in the deracoxib-treated group compared to the placebo control group in all mGCPS behavior categories at most observation times after extubation the day of surgery. The deracoxib group had significantly lower (*P* = 0.0115) posture/activity scores the day of surgery. Analysis of all other mGCPS categories yielded either nonsignificance or nonconvergence of the statistical model. For those dogs that were offered food the day of surgery, 21 of 23 dogs in the deracoxib group were observed to have eaten whereas 14 of 17 dogs in the placebo group ate. No other trends were recognized from the eating data.

Eight of the 31 dogs (26%) receiving deracoxib had an adverse event reported compared to 6 of the 31 dogs (19%) receiving placebo (Table 3). There were no distinct breed, age, or gender predilections and no dogs were removed from the study due to an adverse event. Digestive tract disorders (vomiting, regurgitation, and diarrhea) and postsurgical disorders (abnormal clinical chemistry results) were the most frequently reported adverse events. Two clinically serious adverse events were reported during the study: one report of lameness due to trauma associated with a toe caught on a protruding nail for a dog treated with deracoxib and one report of tachycardia and regurgitation during surgery for a dog treated with placebo. A review of the mean clinical pathology exit results by organ system showed no clinically significant abnormalities in hepatic, renal, or hematological functions.

## 4. Discussion

In the present study, perioperative administration of deracoxib at a dose of 1-2 mg/kg body weight at least 1 h prior to surgery and again for two additional days effectively managed the pain associated with dental surgery in dogs that received butorphanol as a preanesthetic. Dogs enrolled across several clinics were of various breeds and sizes representative of the population of cases expected to be presented to the dental practitioner. Dental extractions that included the creation of a flap, sectioning as necessary, removal of bone, and closure of the surgical site provided an adequate test to assess the analgesic and anti-inflammatory effects of deracoxib in an actual use clinical practice setting. The number of dogs enrolled was considered appropriately powered for collecting meaningful data while minimizing the number of dogs subjected to pain in a rescue study design.

Measuring dental pain is subjective and can be difficult to quantify. A modification of the GCPS scoring system was chosen for this study as the GCPS is a system that assesses multiple aspects of postoperative pain by evaluating spontaneous and evoked behaviors, interactions with people, and clinical observations within a clinical practice setting. The GCPS scoring system allows for assigning a number to behavioral categories that have been shown to provide a descriptive and repeatable assessment of pain [7, 8]. The GCPS was modified to reflect and measure behaviors observed when assessing dogs experiencing pain associated with dental surgery based on consultations with board-certified veterinary dentists. By using the mGCPS scoring system, a degree of consistency was expected that allowed for the adequate evaluation of the response to treatment across the four study sites. This study required that one individual experienced in assessing pain be assigned at each study site to evaluate all dogs at all designated times to reduce the variability in pain assessment scores within a site. Dogs were removed if they scored ≥4 on the mGCPS scoring system or if the pain assessor determined that rescue therapy was needed due to the discomfort of the dog. The intervention cut-off score of “4” is corroborated by previous work that demonstrated a cut-off score of “6” is appropriate when six GCPS categories are evaluated and “5” when “mobility” is not assessed [8]. When the GCPS scoring system was modified for this study, “mobility” and “attention to wound area” were removed and the cut-off score was further reduced to “4”.

While a modification of the GCPS scoring system was used to decrease variability in pain assessments, both for an individual animal response and across different clinical sites, the method still relied on subjective evaluations to measure treatment outcomes. Overall, the scoring system worked well in distinguishing responses between treatment groups; however, a third of the placebo-treated dogs were not rescued. This may have been due to the preanesthetic administration of butorphanol, the scoring system not being sufficiently sensitive enough to detect lower levels of pain in some animals, differences between how dogs interacted with the pain assessor, differences between how pain assessors interpreted the scoring criteria across the different sites, or that some dogs masked their pain. Regardless of the reason, dogs not recognized as painful after procedures that would be expected to cause pain beyond what would be considered manageable by preanesthetic administration of butorphanol alone highlights the need for additional studies and refinement of current pain assessment tools. Pain management, especially in the perioperative period, relies on the practitioner using the most appropriate and effective pain intervention treatments available. Unfortunately, assessment of effective pain management relies on comparisons to treatments that may not have been definitively demonstrated to relieve pain, or comparisons to animals that have not received perioperative pain management (use of a negative control group). The best approach is unclear, but in the absence of a highly sensitive and validated pain assessment tool, the debate should also include consideration for limited “add-on” or negative control studies with rescue treatment.

Dogs requiring pain intervention were typically rescued the day of surgery indicating that administering an NSAID preoperatively is an important pain management practice that may help mitigate the need for more extensive pain medications during and after surgery. This observation is consistent with other studies that assessed the optimal time for administration of perioperative analgesics [17, 23]. Preemptive analgesia cannot eliminate postoperative pain, but can help prevent peripheral and central nervous system sensitization during the surgical procedure thereby reducing the degree of postoperative pain [24]. When comparing mGCPS behavior category scores between treatment groups the day of surgery, a numerical improvement was observed as early as 90 min after extubation that continued to benefit the surgical patient throughout the day of surgery. Subsequent treatments once daily for 2 days after dental surgery sustained the clinical benefit as evident by only one deracoxib-treated dog being rescued the day after surgery whereas three dogs in the placebo control group were rescued the day after surgery and one was rescued the second day after surgery.

Deracoxib appeared to be well tolerated in dogs when used at 1-2 mg/kg for up to 3 days to manage postoperative pain and inflammation associated with dental surgery. Deracoxib at the recommended dose was shown to be safe in target animal safety studies [25], and clinical experience has shown that, when used as directed, deracoxib is both safe and effective for managing postsurgical orthopedic pain and pain associated with osteoarthritis. Digestive tract disorders (e.g., primarily vomiting) were the most frequently reported adverse events and are typical for the NSAID class of drugs [26, 27]. There were no reports of excessive bleeding during or after the dental surgery. As with all NSAIDs, dogs should be well hydrated and undergo a thorough history and physical examination prior to initiation of any NSAID therapy.

Results indicate perioperative administration of deracoxib to dogs at 1-2 mg/kg/day for 3 days is well tolerated and significantly improves analgesia in the postoperative surgical period following dental extractions.

## Figures and Tables

**Table 1:** Modified Glasgow Composite Pain Scale (mGCPS) behavioral assessment categories and scoring based on the severity of the behavior or response observed.

Behavior category	Score	Descriptor
Vocalization	[0]	Quiet
	[1]	Whimpering or crying
	[2]	Groaning
	[3]	Screaming

Response to touch	[0]	Do nothing
	[1]	Looks around
	[2]	Flinch
	[3]	Growl or guard area
	[4]	Snap
	[5]	Cry

Demeanor	[0]	Happy and content and bouncy
	[1]	Quiet
	[2]	Indifferent or nonresponsive to surroundings
	[3]	Nervous, anxious or fearful
	[4]	Depressed or nonresponsive to stimulation

Posture/activity	[0]	Comfortable
	[1]	Unsettled
	[2]	Restless
	[3]	Hunched or tense
	[4]	Rigid

Eating*	[0]	Eating normally
	[1]	Eating more slowly
	[2]	Eating with reluctance
	[3]	Not eating

*Eating was not included in score summaries for determining pain intervention.

**Table 2:** Comparison of treatment success and failure rates between the deracoxib and placebo treatment groups based on dogs requiring pain intervention treatment.

Treatment group	Treatment outcome		*P* value	Odds ratio	Confidence interval	
	Success	Failure			Lower	Upper
Deracoxib (*n* = 27)	23 (85.2%)	4 (14.8%)	0.0006	11.5	3.1	42.4
Placebo (*n* = 30)	10 (33.3%)	20 (66.7%)				

**Table 3:** Frequency and distribution of adverse clinical observations reported for all dogs enrolled in the study, including adverse clinical observations reported after intervention therapy and observations reported by the owners after the dog returned home.

Clinical observation	Number of dogs with reported adverse clinical observations*	
	Deracoxib *n* = 31	Placebo *n* = 31
Vomiting	4	1
Regurgitation	0	2
Diarrhea/soft stool	3	1
Increased AST^†^	3	0
Increased ALT^†^	1	0
Hematuria	1	0
Leukocytosis	1	1
Neutrophilia	1	1
Lameness	1	0
Facial swelling	0	1
Tachycardia	0	1

*Dogs may have experienced more than one of the observations during the study.

^†^Includes animals with results over 2x the high normal.
